# Healing Rates in a Multicenter Assessment of a Sterile, Room Temperature, Acellular Dermal Matrix Versus Conventional Care Wound Management and an Active Comparator in the Treatment of Full-Thickness Diabetic Foot Ulcers

**Published:** 2016-09-07

**Authors:** Geoffrey L. Robb, Geoffrey C. Gurtner

**Affiliations:** ^a^Department of Plastic Surgery, University of Texas MD Anderson Cancer, Houston; ^b^Department of Surgery, Stanford University, Stanford, Calif

**Keywords:** acellular dermal matrix, biologic mesh, wounds, breast reconstruction, tissue expander

Dear Editor,

We read with interest your article, titled “Healing Rates in a Multicenter Assessment of a Sterile, Room Temperature, Acellular Dermal Matrix Versus Conventional Care Wound Management and an Active Comparator in the Treatment of Full-Thickness Diabetic Foot Ulcers,*”* published in the February 2016 of *ePlasty*. As plastic surgeons with reconstructive practices spanning a variety of disease states (cancer, trauma, wounds), we are always interested in rigorous studies that improve the evidence base in clinical care. As you know, human acellular dermal matrices (ADMs) are very widely used in a variety of settings and are a mainstay for many of us in tissue expander–based breast reconstruction. We believe any study that demonstrates a significant difference in the clinical outcomes of different products is important.

While the promotional materials for different products always claim that there are big differences between product “X” and product “Y,” there is very little clinical evidence to support this claim. The Plastic Surgery literature has very few studies that compare different ADMs “head-to-head.” The few trials that do exist are small and underpowered to detect any differences. In contrast, the current DermACELL trial is a large, rigorous head-to-head comparison, which provides plastic surgeons with some new and important insights. In this article, Walters and colleagues demonstrate superior healing rates in terms of both wound closure and reduction in wound size as compared with both conventional care and GraftJacket.^1^ Although not all differences were statistically significant, many were and the magnitude of the differences is difficult to ignore. Although the product GraftJacket is probably not familiar to most plastic surgeons, it is simply the market leader, Alloderm, rebranded and relabeled for sales to wound centers. It is procured, processed, and sold by the parent company of LifeCell under a different name.

The authors are to be congratulated for conducting the largest and most rigorous study examining ADMs in a head-to-head fashion. It is the first study to demonstrate a clinical benefit of using one product over another. It puts to rest the viewpoint that all ADMs are alike in terms of clinical performance. In our view, these data are of direct relevance to our breast reconstruction practices, because the identical DermACELL product is also used in postmastectomy breast reconstruction. In both wounds and breast reconstruction, an ADM is used to replace lost or weakened tissue and the success of the product depends on rapid incorporation. It is logical to assume that since the underlying biology is the same, that superior performance in one setting (ie, wounds) would translate into superior performance in another (ie, breast reconstruction). It is hoped that with further study, we will obtain a more nuanced view of the relative merits of all the different products currently marketed to plastic surgeons. For now, the take-home message is that not all ADM products are alike and that some seem to perform better than others. The next challenge for us is to understand “why.”
